# Hepatitis B Virus-Induced Resistance to Sorafenib and Lenvatinib in Hepatocellular Carcinoma Cells: Implications for Cell Viability and Signaling Pathways

**DOI:** 10.3390/cancers16223763

**Published:** 2024-11-08

**Authors:** Narmen Esmael, Ido Lubin, Ran Tur-Kaspa, Romy Zemel

**Affiliations:** 1 Molecular Hepatology & Transplantation Immunology Research Labs, Felsenstein Medical Research Center, Tel-Aviv University, Beilinson Campus, Rabin Medical Center, Petah Tikva 4941492, Israel; narmenesmael@mail.tau.ac.il (N.E.); idolu@tauex.tau.ac.il (I.L.); rturkaspa@clalit.org.il (R.T.-K.); 2 Liver Institute, Beilinson Campus, Rabin Medical Center, Petah Tikva 4941492, Israel; 3 Azrieli Faculty of Medicine, Bar-Ilan University, Safed 1311502, Israel

**Keywords:** HBV, sorafenib, lenvatinib, resistance

## Abstract

**Background/Objectives**: Sorafenib and lenvatinib are tyrosine kinase inhibitors used in hepatocellular carcinoma (HCC) treatment. This study investigates how hepatitis B virus (HBV) infection affects their efficacy in HepG2 hepatoma cells. **Methods**: HepG2 and HBV-infected HepG2/2215 cells were treated with varying concentrations of both drugs. The cell viability, cell cycle gene expression, cycle progression, and phosphorylation levels of ERK and AKT were analyzed. Results: The HBV-infected cells showed significant alterations in their cell cycle gene expressions, with an 80-fold increase in CCND2 expression and a higher proportion of cells in the G2/M phase, indicating enhanced proliferation. While both drugs decreased HepG2 cell viability in a concentration-dependent manner, HBV infection conferred resistance, as evidenced by the increased viable cells in the HepG2/2215 cultures. Sorafenib and lenvatinib decreased key cyclin and cyclin-dependent kinase expressions in uninfected cells, with less effect on the HBV-infected cells. Both drugs lowered the pERK and pAKT levels in the HepG2 cells. In the HBV-infected cells, sorafenib reduced the pERK and pAKT levels to a lesser extent. However, treatment with lenvatinib elevated the levels of pERK and pAKT. **Conclusions**: In conclusion, HBV infection increases resistance to both sorafenib and lenvatinib in hepatoma cells by influencing the cell cycle regulatory genes and critical signaling pathways. However, the resistance mechanisms likely differ between the two medications.

## Simple Summary

In real-life applications, lenvatinib has been found to have a better tumor response than sorafenib in patients with HBV-related HCC, showing that treatment with lenvatinib may be useful in individuals with HBV infections. However, in this study, we show that HBV confers resistance to lenvatinib at an even deeper level than its resistance to sorafenib. It is highly likely that the mechanisms of resistance to these two medications are distinct. Our findings may explain why lenvatinib and sorafenib have had equal effects on overall survival and progression-free survival in patients with HBV-related HCC, despite the apparent potential benefit of sorafenib. However, additional research is required to identify the processes that drive this resistance and to develop more effective therapies for HBV-associated HCC. These findings highlight the need for tailored therapeutic strategies to overcome HBV-induced resistance in HCC treatment.

## 1. Introduction

Hepatocellular carcinoma (HCC) is one of the major causes of cancer-related death worldwide, and chronic hepatitis B (HBV) infection is a major etiological factor responsible for about 50% of cases worldwide [1,2]. Therefore, effective therapeutic interventions against HCC in relation to HBV infection are of paramount importance.

Sorafenib and lenvatinib are two multi-kinase inhibitors approved for the treatment of advanced HCC. Sorafenib exerts its anti-proliferative and anti-angiogenic action on tumor cells through RAF, VEGFR, PDGFR, and KIT kinases. Clinical trials have documented that it can prolong the survival of patients with HCC, thus becoming a standard of care in patients with advanced HCC [3,4]. Lenvatinib is a novel multi-kinase inhibitor targeting VEGFR1-3, FGFR1-4, PDGFRα, RET, and KIT [5]. In some clinical studies, it has been demonstrated to be non-inferior to sorafenib [6,7], while in another study, it was reported to lead to better response rates and progression-free survival than sorafenib [8]. Both sorafenib and lenvatinib are both confronted with the major problem of drug resistance, which limits their long-term effectiveness. These resistances include genetic mutations, the activation of alternative signaling pathways, and alterations in the tumor microenvironment [9,10,11].

HBV infection worsens the problem of drug resistance in HCC. The virus integrates into the genome of the host and thus influences multiple cellular events controlling drug metabolism and cell survival [12]. HBV activates the RAF-MEK-ERK and PI3K-AKT pathways, which are also targets of sorafenib and lenvatinib, and may, hence, induce cross-resistance. Studies have shown that HBV-infected cells have acquired resistance to sorafenib, in part due to the influence of the virus on those signaling pathways and by its impact on the expression of cell cycle regulatory genes [13,14,15]. The efficacy of lenvatinib in HBV-positive HCC patients is still debatable. A recent real-world study comparing lenvatinib and sorafenib as first-line systemic therapies for unresectable HCC in chronic HBV patients yielded mixed results. While lenvatinib demonstrated better tumor response compared to sorafenib, it showed equal overall survival (OS), time to progression (TTP), and progression-free survival (PFS) [8]. The observation that the sorafenib group had achieved OS comparable to the lenvatinib group was explained by the fact that more frequent subsequent anti-HCC treatments were received by the former. Although this observation may explain the comparable outcomes, it also opens up the avenue for how HBV may influence treatment efficacy.

In the current study, we aimed to investigate the influence of HBV infection on the efficacy of sorafenib and lenvatinib in HepG2 hepatoma cells. In this respect, the mechanisms of HBV-mediated resistance against these therapies have been pursued through research on cell viability, gene expression in the cell cycle process, and the modulation of key signaling pathways. Understanding the mechanisms by which cancer cells acquire resistance may offer valuable insights that could guide future treatment strategies for HBV-associated HCC and improve patient outcomes.

## 2. Materials and Methods

### 2.1. Cell Lines and Treatments

HepG2 (RRID:CVCL_0027) and HepG2/2215 cells (CVCL_L855) [16] were grown in DMEM medium containing 4.5 g/L of D-glucose, 10% fetal bovine serum (FBS), 0.1 mg/mL of penicillin/streptomycin (Biological Industries, Beit-Haemek, Israel), and 2 mM of glutamine (Biological Industries, Israel) and incubated at 37 °C. The cells were plated in 96-well plates (3 × 10^4^ cells/well) or 6-well plates (1.2 × 10^6^/well) and treated with sorafenib (Selleck Chemicals, Houston, TX, USA) or lenvatinib (Cayman Chemical, Ann Arbor, MI, USA) at different concentrations (0–12 µM) for 24 h.

### 2.2. Crystal Violet Cytotoxicity Assay

The cells were stained with 0.5% crystal violet solution (Sigma-Aldrich, Rehovot, Israel). The cell viability was detected using the crystal violet staining protocol, with solubilization of the dye adsorbed by the cells using 1% SDS. The optical density was measured at 570 nm for quantification.

### 2.3. Alamar Blue Cell Viability Assay

Cell proliferation was determined by Alamar Blue™ Cell Viability Reagent (Invitrogen, Eugene, OR, USA). The reagent was added to the culture medium (final dilution of 1:10), and the fluorescence (540 nm/590 nm) was measured after 1–2 h. The experiments were performed in triplicate.

### 2.4. Real-Time PCR

The total RNA was isolated using a miniprep RNA isolation kit (A&A Biotechnology, Gdańsk, Poland) according to the manufacturer’s protocol. The extracted RNA was stored at −80 °C until use.

CDNA synthesis was performed from 1 μg RNA using qScript reverse transcriptase (Quantabio, Beverly, MA, USA). The DNA and RNA concentrations were quantified using the Nano-Drop spectrophotometer (Thermo Fisher Scientific, Waltham, MA, USA). For real-time PCR analysis, SYBR green technology was employed. The reaction containing a 5× reaction mixture and cDNA in 10 μL of the total reaction volume was performed in triplicate. Gene-specific PCR primers for the SYBER assays were obtained from Hylabs (Rehovot, Israel). The RNA gene *RSP11* was used as the reference gene. The primer sequences are listed in Table 1.

### 2.5. Cell Cycle Analysis by Flow Cytometry

The cell cycle status was analyzed by flow cytometry. The cells were cultured in a 6-well plate, treated with sorafenib or lenvatinib for 24 h, and then washed by PBS X2 centrifugation at 1000 rpm for 5 min. at 4 °C. The cell pellet was suspended in 0.5 cold PBS and fixed by adding 4.5 mL of 70% cold ethanol dropwise, with gentle vortexing. The cells were then incubated for >2 h at −20 °C, washed with PBS, and centrifuged at 1000 rpm for 5 min 4 °C. Staining with propidium iodide (P4170, Sigma-Aldrich, Rehovot, Israel) was conducted just prior to the flow cytometry. The cell pellet was resuspended in 0.5 mL PBS containing 0.025 mL of PI (50 μg/mL in PBS and 5 μL of 100 μg/mL DNase-free RNase (Sigma-Aldrich, Rehovot, Israel). The cells were then incubated at 37 °C for 15 min. The samples were analyzed on a Beckman–Coulter Gallios apparatus. The parameters were adjusted for the measurement of single cells using forward and side scatter plots.

### 2.6. Western Blot Analysis

The cells were homogenized with RIPA lysis buffer containing protease (Sigma Aldrich, Rehovot, Israel) and phosphatase (Roche) inhibitor cocktails. The protein concentrations were determined using the Bradford protein assay (Bio-Rad Laboratories, Hercules, CA, USA). The lysates were resolved by SDS–PAGE through 4–12% precast polyacrylamide gel (Bio-Rad Laboratories, Hercules, CA, USA) and transferred to a nitrocellulose membrane. The membranes were blocked for 1 h in blocking buffer (Bio-Rad Laboratories, Hercules, CA, USA), and then incubated with specific antibodies overnight at 4 °C. After washing (X3 PBS), a secondary antibody was applied at room temperature for one hour. Anti-tublin was used as a loading control and run on the same blot as the experimental samples. The results were visualized using the iBright system.

### 2.7. Statistical Analysis

The experiments were performed at least three times. Student’s two-tailed *t*-test for independent data was performed using GraphPad Prism (RRID:SCR_002798). The results were considered significant when *p* < 0.05.

## 3. Results

### 3.1. Effects of Sorafenib and Lenvatinib on Cell Viability

**Figure 1:**
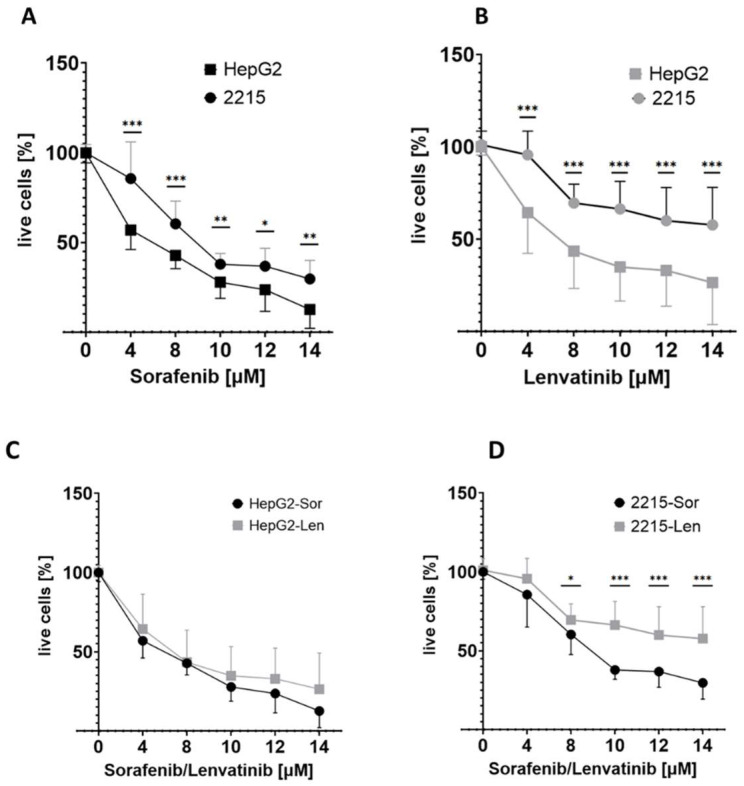
Sorafenib and lenvatinib treatment decreased the viability of HepG2 cells. HepG2 cells and HepG2/2215 HBV-infected cells (2215) were exposed to varying concentrations (0 to 14 µM) of (**A**) sorafenib or (**B**) lenvatinib for 24 h and analyzed for viability by AlamarBlue. A comparison of the effects of sorafenib and of lenvatinib in (**C**) HepG2 cells and in (**D**) HepG2/2215 cells is shown. The percentage of live cells was determined compared to the non-treated cells, set as 100%. The data are presented as the means ± standard deviations. *n* = 3. * *p* < 0.05, ** *p* < 0.005, and *** *p* < 0.0005.

To evaluate the impact of HBV infection on the effectiveness of sorafenib and lenvatinib, we initially examined the effects of these agents on the viability of HepG2 hepatoma cells, which are uninfected by HBV. The HepG2 cells were treated with varying concentrations of sorafenib and lenvatinib for 24 h, and their cell viability was assessed using the AlamarBlue reagent. Both sorafenib and lenvatinib demonstrated a concentration-dependent decrease in the viable cell numbers (Figure 1), indicating their cytotoxic effects on hepatoma cells. There were no significant differences in the efficacy of these two drugs (Figure 1C). To investigate the effects of HBV infection on the efficacy of sorafenib and lenvatinib, we used HepG2/2215 cells, which were derived from HepG2 cells transfected with HBV DNA. As presented in Figure 1, an increase in the viable cell numbers in the presence of HBV was observed, suggesting that HBV infection conferred resistance to both chemotherapy drugs.

Based on the initial viability results, we selected a concentration of 10 µM for both sorafenib and lenvatinib to further investigate the effects of HBV on chemotherapy resistance. At this concentration, similar increases in viable cells (28% and 30%, respectively (Figure S1) were observed when treating the HBV-expressing cells. These findings were corroborated by crystal violet assays, which also indicated increases in the cell numbers upon treatment with either drug in the presence of HBV (Figure S1B).

### 3.2. Effects of Sorafenib and Lenvatinib on Cell Cycle

**Figure 2:**
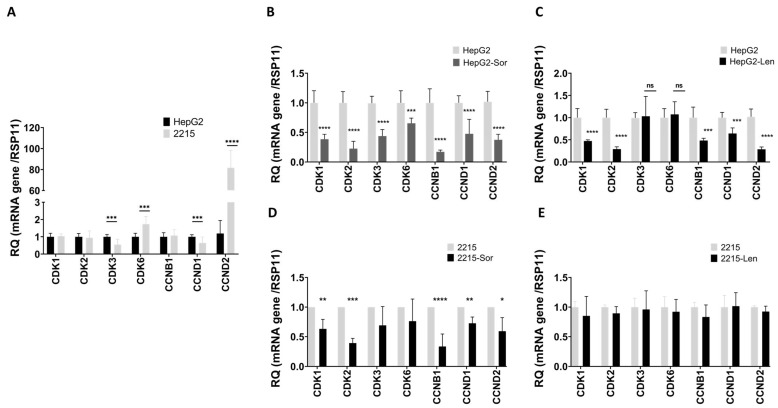
The effects of sorafenib and lenvatinib treatments on the expressions of cell cycle genes in the HepG2 and HepG2/2215 cell lines. QRT-PCR of cell cycle gene expression; *CDK1*, *CDK2*, *CDK3*, *CDK6*, *CCNB1*, *CCND1*, and *CCND2*. (**A**) HepG2 and HepG2/2215 cell lines without treatment; (**B**) HEPG2 cells treated with sorafenib (Sor) and (**C**) lenvatinib (Len); (**D**) HepG2/2215 cells treated with sorafenib and (**E**) lenvatinib. The results are shown as the relative quantity normalized to the RPLP11 mRNA values. The data are presented as the means ± standard deviations; *n* = 3. * *p* < 0.05, ** *p* < 0.005, *** *p* < 0.0001 and **** *p* < 0.00005.

To determine if the inhibition of cell proliferation by sorafenib and lenvatinib was associated with cell cycle dysregulation, we analyzed the expression levels of cell cycle regulatory genes in HepG2 and HepG2/2215 cells using qRT-PCR. In the absence of drug treatment, HBV infection caused a substantial increase in cyclin D2 (CCND2) levels, a regulatory protein crucial for G2/M phase transition, with an 80-fold increase compared to the uninfected HepG2 cells (Figure 2). There was also a significant decrease in cyclin D1 (CCND1) expression and an elevation of cyclin-dependent kinase 6 (CDK6), which promotes the G1/S transition.

The treatment of HepG2 cells with either sorafenib or lenvatinib led to a noticeable decrease in the expressions of several cyclin-dependent kinases, including CDK1, CDK2, CCNB1, CCND1, and CCND2. The CDK6 levels remained largely unchanged under both treatments (Figure 2B,C). In HBV-harboring cells, the sorafenib treatment resulted in a similar downregulation of cell cycle-regulating genes (Figure 2D). However, lenvatinib treatment in HBV-infected cells had no significant effect on the expression levels of the cell cycle regulating genes (Figure 2E).

**Figure 3:**
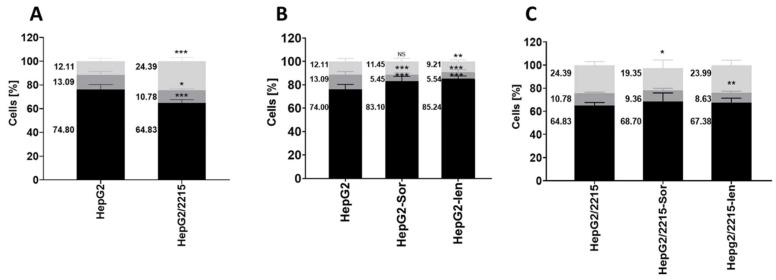
The effect of sorafenib (Sor) and lenvatinib (Len) on cell cycle phases in HepG2/2215 cell line. Stacked bar graphs showing the percentage of cells in different phases of the cell cycle. Flow cytometry results shown distribution DNA content: G1 (black), S (dark gray), G2/M (light gray) phases in (**A**) HepG2 and HepG2/2215 cell line without chemotherapeutic treatments (**B**) HepG2 cells ± sorafenib and lenvatinib treatment (**C**) HepG2/2215 cells ± sorafenib and lenvatinib treatment. Data of three experiments are presented as mean ± standard deviation (numbers at the left side of the bars represent the mean of % cell number). *NS*—non significant, * *p* < 0.05, ** *p* < 0.005, *** *p* < 0.001.

Cell cycle progression was further assessed by analyzing the DNA content through propidium iodide staining and flow cytometry. As expected, the HepG2/2215 cells showed a significantly higher proportion of cells in the G2/M phase (24.39%) compared to the HepG2 cells (12.11%), indicating increased proliferation rates (Figure 3A). The treatment of the HepG2 cells with either sorafenib or lenvatinib (Figure 3B) significantly reduced the S phase (13.09% vs. 5.45% and 5.55%, respectively). In addition to the effect on the S phase, lenvatinib caused a decrease in the G2/M phase (13.1% vs. 9.2%). In the HBV-infected cells, both drugs had a smaller effect on cell cycle progression. The lenvatinib treatment reduced the S phase (10.78% vs. 8.6%), while the sorafenib treatment decreased the G2/M phase (24.39% vs. 19.35%) (Figure 3C).

These results indicate that sorafenib and lenvatinib inhibited cell cycle progression in uninfected HepG2 cells. However, lenvatinib had a more pronounced impact on cell cycle progression compared to sorafenib in the HepG2 cells. Resistance to the drug treatment acquired by HBV has an impact on both drugs, with distinct mechanisms affecting cell cycle progression and gene expression.

### 3.3. The Effect of Sorafenib and Lenvatinib on PERK Expression

Recent studies have shown that HBV infection induces resistance to sorafenib [13,14,15]. However, there are no publication indicating the role of HBV in lenvatinib resistance. The MEK/ERK pathway, which is critical for cell proliferation, is frequently implicated in both sorafenib [17,18,19] and lenvatinib resistance [20,21,22,23,24].

**Figure 4:**
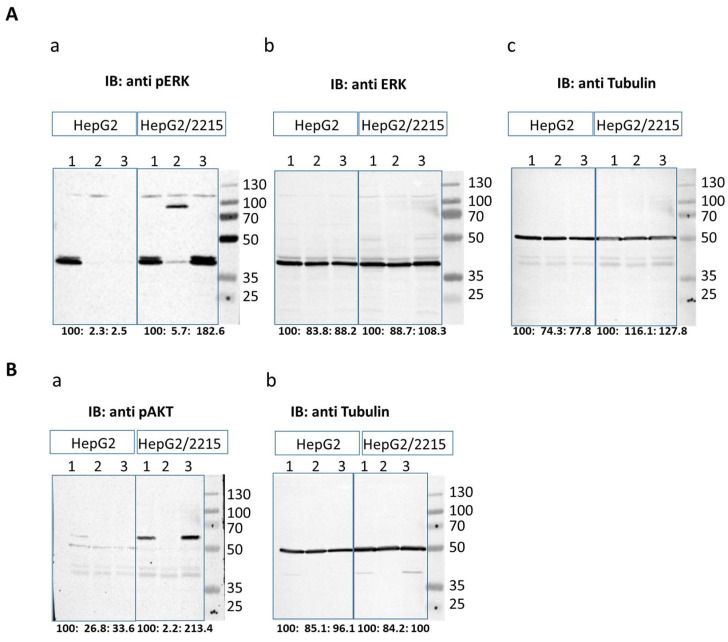
PERK and pAKT expression levels following treatment with sorafenib and lenvatinib. HepG2 and HepG2/2215 cells were treated with 10 µM sorafenib or 10 µM lenvatinib for 24 h, and the proteins were extracted and analyzed by Western blot for the protein levels of (**A**) pERK (**a**), ERK (**b**) and tubulin (as the internal control) (**c**) and for (**B**) pAKT (**a**) and tubulin (**b**). iBright analysis was used to quantify intensity, and the percentage of relative intensity, normalized to untreated cells, is displayed at the membrane’s bottom.

It is suggested that the MEK/ERK pathway is a central player in resistance development in HCC. Therefore, we tested the possible involvement of this pathway in HBV mediated lentanivib resistance. We investigated the effect of sorafenib and lenvatinib on the phosphorylation of ERK (pERK). HepG2 and HepG2/2215 cells were cultured with 10 µM sorafenib or lenvatinib for 24 h, and pERK expression was assessed by Western blot. Treatment with either drug resulted in a decrease in pERK levels in HepG2 cells (Figure 4A). In HBV-harboring hepatoma cells, treatment with sorafenib resulted with a reduction in pERK levels, although to a lesser extent than in HEPG2 cells. In contrast, lenvatinib treatment remarkably increased pERK levels in HBV-infected cells.

Lenvatinib, block kinases receptors and ultimately inhibits the Ras-Raf-ERK and PI3K-AKT pathways. Resistance to lenvatinib was shown to stimulate PI3K/AKT and MAPK/ERK signaling pathways [25]. The high elevation in pERK prompt us to test whether AKT pathway is also affected by the drugs treatments in HBV infected cells. In HepG2 cells, treatment with either sorafenib and lenvatinib reduced pAKT levels (Figure 4B). In HBV-infected cells, sorafenib similarly reduced pAKT levels, whereas lenvatinib treatment resulted in a significant increase in pAKT levels.

Overall, these findings suggest that while sorafenib and lenvatinib effectively inhibit key signaling pathways in uninfected hepatoma cells, HBV infection alters their impact, potentially contributing to drug resistance through distinct mechanisms involving PERK and pAKT signaling pathways.

## 4. Discussion

Our findings indicate that while sorafenib and lenvatinib are effective in reducing cell viability and disrupting cell cycle progression in uninfected hepatoma cells, HBV infection confers resistance to these drugs through the differential modulation of key signaling pathways. The distinct effects on cell cycle-related gene expression and the PI3K/AKT and MAPK/ERK signaling pathways highlight the complex mechanisms by which HBV mediates drug resistance.

These findings are particularly relevant given the recent systematic review and meta-analysis [26], which demonstrated comparable overall survival between sorafenib and lenvatinib in HCC patients, though without specific consideration of their HBV status.

Our study demonstrated that both sorafenib and lenvatinib decreased the viability of HepG2 hepatoma cells in a concentration-dependent manner. This finding aligns with previous research highlighting the efficacy of sorafenib and lenvatinib as tyrosine kinase inhibitors that induce cell death in various cancer cell lines, including HCC cells [4,6]. The absence of significant differences in efficacy between the two drugs in uninfected cells suggests that both TKIs target similar pathways to exert their cytotoxic effects.

Treatment with sorafenib or lenvatinib inhibits cell cycle progression by reducing the S and G2/M phases in uninfected HepG2 cells, highlighting their efficacy in arresting cell proliferation. qRT-PCR analysis of the uninfected hepatoma cells revealed a reduction in *CDK1*, *CDK2*, *CCNB1*, *CCND1*, and *CCND2* expression upon treatment with sorafenib or lenvatinib, suggesting that these drugs can effectively disrupt cell cycle progression, corroborating previous reports on the mechanisms of TKIs in inhibiting cell proliferation [4,6].

HBV is known to modulate cellular pathways to promote survival and resistance to chemotherapy [13,14,15,27,28]. Indeed, qRT-PCR analysis revealed significant alterations in cell cycle gene expressions upon HBV infection. It showed a substantial increase in *CCND2* expression and a decrease in *CCND1*, indicating a shift in cell cycle regulation that favored proliferation. Flow cytometry analysis of the DNA content revealed significant differences in the cell cycle phases between the HepG2 and HepG2/2215 cells. The HBV-infected cells exhibited a higher proportion of cells in the G2/M phase, indicative of enhanced proliferation. This observation is in line with findings that HBV infection can accelerate cell cycle progression and increase cell proliferation [28,29].

Previous studies have already shown that HBV infection confers resistance to sorafenib treatment [13,14,27]. In this study, we showed that HBV infection also confers resistance to lenvatinib, as indicated by an increase in viable HepG2/2215 cells compared to uninfected cells. Sorafenib treatment in HBV-harboring cells resulted in the downregulation of cell cycle-regulating genes, similar to the effect observed in the non-infected cells, but it had no significant effect on *CDK3* or *CDK6* expression. Although both sorafenib and lenvatinib showed similar decreases in cell death in HBV-infected cells, indicating drug resistance, lenvatinib had no significant effect on any of the cell cycle-regulating genes tested and no effected on cell cycle progression. This differential impact on the cell cycle phases and gene expression imply that HBV may employ different strategies to counteract the cytostatic effects of sorafenib and lenvatinib.

Consistent with their roles in targeting the PI3K/AKT and the MAPK/MEK/ERK signaling pathways, both sorafenib and lenvatinib [4,25] reduced the pERK and pAKT levels in the HepG2 cells. HBV is known to activate various survival pathways, including the PI3K/AKT and MAPK/ERK pathways, which can contribute to the reduced efficacy of TKIs and to increased cell survival and resistance to apoptosis [29]. In our study, the sorafenib treatment in the presence of HBV resulted in reduced pERK levels, although to a lesser extent than in uninfected cells, while lenvatinib significantly increased pERK levels. A similar effect was obtained with the pAKT levels. This observation suggests that HBV infection may differentially modulate the MAPK/MEK/ERK and the PI3K/AKT pathways to mediate resistance in response to sorafenib and lenvatinib. Of note, our experimental concentrations differed from clinically achievable plasma concentrations, which are typically 250 ng/mL and 350 ng/mL for lenvatinib [30] and 5 to 10 μg/mL for sorafenib [31]. However, our experimental design deliberately employed equimolar concentrations to evaluate the direct cellular effects of these tyrosine kinase inhibitors. This methodological approach facilitates the assessment of their molecular mechanisms at the cellular level, independent of pharmacokinetic variables. This provides valuable insights into target engagement and pathway modulation. This standardized in vitro methodology establishes a foundation for subsequent in vivo investigations, potentially informing both combination therapeutic strategies and future drug development initiatives. While our study provides detailed mechanistic insights into the comparative effects of sorafenib and lenvatinib, we acknowledge that the use of a single cell line (HepG2) represents a limitation. Although HepG2 is a well-established model for HCC research, cellular responses might vary across different HCC cell lines. Future studies incorporating multiple cell lines would be valuable to validate the broader applicability of our findings.

## 5. Conclusions

In real-life applications, lenvatinib has been found to have a better tumor response compared to sorafenib in patients with HBV-related HCC [6,8,25]. Nevertheless, we have demonstrated that in an in vitro cell culture system, HBV also confers resistance of lenvatinib. Remarkably, the resistance to lenvatinib is more profound than to sorafenib treatment. It seems that the mechanism of resistance is different between these two drugs and may involve the MAPK/MEK/ERK and the PI3K/AKT pathways. To be clear, the experimental concentrations used in our study differ from clinically achievable plasma concentrations, which limits the direct clinical applicability of our findings. Future studies should concentrate on drug concentrations that are clinically relevant. Although lenvatinib may appear to be superior to sorafenib for patients with HBV-related HCC, our research may help to explain why lenvatinib and sorafenib have comparable effects on overall survival and progression-free survival.

## Figures and Tables

**Table 1:** Primer sequences for qRT-PCR of specific genes.

Accession Number	Gene Name	Sequence (5′ → 3′)	Product Size (bp)
NM_001786.5	*CDK1*	CCTGGTCAGTACATGGATTCTT	150
		AGCCAGTTTAATTGTTCCTTTGTC	
NM_001798.5	*CDK2*	AGAAACAAGTTGACGGGAGAG	136
		GCAGCTTGACAATATTAGGATGG	
NM_001145306.1	*CDK6*	CCGAAGTCTTGCTCCAGTC	149
		GAGTCCAATCACGTCCAAGAT	
NM_031966.4	*CCNB1*	GGCTTTCTCTGATGTAATTCTTGC	142
		GTATTTTGGTCTGACTGCTTGC	
NM_004701.4	*CCNB2*	GGCTGGTACAAGTCCACTCC	107
		CTTCTTCCGGGAAACTGGCT	
NM_053056.3	*CCND1*	CATCTACACCGACAACTCCATC	135
		TCTGGCATTTTGGAGAGGAAG	
NM_001759.4	*CCND2*	ACTTGTGATGCCCTGACTG	141
		ACTTGGATCCGTCACGTTG	
NM_001015.5	*RPS11*	CTACAAGAACATCGGTCTGGG	150
		ATGGTCCTCTGCATCTTCATC	

## Data Availability

The original contributions presented in the study are included in the article/Supplementary Materials. Further inquiries can be directed to the corresponding author.

## Supplementary Materials

The following supporting information can be downloaded at: https://www.mdpi.com/article/10.3390/cancers16223763/s1, Figure S1: Resistance to Sorafenib and to Lenvatinib in the presence of HBV. Cells treated for 24 h under 10 µM Sorafenib or 10 µM Lenvatinib. The percentage of live cells was assessed by (**A**) AlamarBlue assay and (**B**) Crystal violet stained cells light microscopic images (at 10×), (**C**) quantification by eluted stain (OD 570 nm). Data are presented as mean ± standard deviation n = 3. *ns*—non significant.
