# Supplementary material for: Hepatitis B Virus-Induced Resistance to Sorafenib and Lenvatinib in Hepatocellular Carcinoma Cells: Implications for Cell Viability and Signaling Pathways
Source: Cancers (Basel). 2024 Nov 8;16(22):3763. doi: 10.3390/cancers16223763 (PMC11592932; doi:10.3390/cancers16223763)
Supplement: Supplementary file 1 [file cancers-16-03763-s001.zip › cancers-3259935-supplementary.pdf]

Supplementary Materials:.

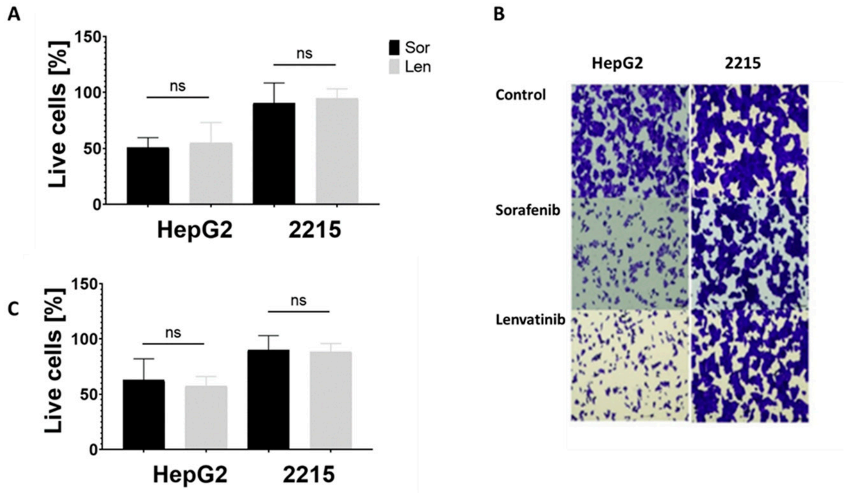

Figure S1. Resistance to Sorafenib and to Lenvatinib in the presence of HBV. Cells treated for 24 h under 10 μM Sorafenib or 10 μM Lenvatinib. The percentage of live cells was assessed by (**A**) AlamarBlue assay and (**B**) Crystal violet stained cells light microscopic images (at 10 ×), (**C**) quantification by eluted stain (OD 570 nm). Data are presented as mean ± standard deviation n = 3. *ns*- non significant.
